# Pathologic abnormalities of deep placentation in the great obstetrical syndromes: Implications for understanding the pathophysiology, risk assessment in early pregnancy, and personalized prevention

**DOI:** 10.1016/j.jri.2026.104846

**Published:** 2026-01-29

**Authors:** Gergő Leipold, Roberto Romero, Pierre-Yves Robillard, Petra Merkely, Máté Posta, Gábor Szalai, Zsófia Benkő, Tamás Marton, Sándor Valent, Adi L. Tarca, Beáta Hargitai, Sándor Nagy, Zoltán Papp, Offer Erez, Nándor Ács, Nándor Gábor Than

**Affiliations:** a Department of Obstetrics and Gynecology, School of Medicine, Semmelweis University, Budapest, Hungary; b Pregnancy Research Branch, NICHD/NIH/DHHS**, Bethesda, MD, USA; c Department of Obstetrics and Gynecology, University of Michigan, Ann Arbor, MI, USA; d Department of Epidemiology and Biostatistics, Michigan State University, East Lansing, MI, USA; e Service de Néonatologie, Centre Hospitalier Universitaire Sud Réunion, La Réunion, France; f Centre d’Etudes Périnatales Océan Indien, Centre Hospitalier Universitaire Sud Réunion, La Réunion, France; g Systems Biology of Reproduction Research Group, Institute of Molecular Life Sciences, HUN-REN Research Centre for Natural Sciences, Budapest, Hungary; h Doctoral College, Károly Rácz Conservative Medicine Division, Semmelweis University, Budapest, Hungary; i Department of Surgery, School of Medicine, University of Pécs, Pécs, Hungary; j Genesis Theranostix Group, Budapest, Hungary; k 1st Department of Pathology and Experimental Cancer Research, School of Medicine, Semmelweis University, Budapest, Hungary; l Department of Obstetrics and Gynecology, School of Medicine, Wayne State University School of Medicine, Detroit, MI, USA; m Center for Molecular Medicine and Genetics, Wayne State University, Detroit, MI, USA; n Department of Obstetrics and Gynecology, Széchenyi István University, Győr, Hungary; o Maternity Private Clinic of Obstetrics and Gynecology, Budapest, Hungary; p Department of Obstetrics and Gynecology, Soroka University Medical Center, Ben Gurion University of the Negev, Be'er Sheva, Israel

**Keywords:** Atherosis, Biomarkers, Deep placentation, Failure of physiological transformation, Liquid biopsy, Personalized medicine, Placenta Growth Factor, Prenatal diagnosis, Screening, Soluble endoglin, Soluble Fms-like tyrosine kinase 1, Trophoblast invasion

## Abstract

The concept of the *Great Obstetrical Syndromes* was introduced to explain the unique nature of obstetrical disease, which differs fundamentally from disorders in other areas of medicine. These syndromes, including preeclampsia, fetal growth restriction, fetal death, and spontaneous preterm birth, represent clinical endpoints rather than single diseases and share defining characteristics: they arise from multiple etiologies, have a prolonged subclinical phase, involve the fetus as an active participant, are adaptive in nature, and result from complex genetic and environmental interactions between the mother and fetus. Among the diverse mechanisms leading to these syndromes, abnormalities of the maternal supply line to the placenta constitute one major etiology and are often caused by vascular disorders affecting the maternal cardiovascular system and uterine spiral arteries, resulting in placental lesions of maternal vascular malperfusion. The most severe spiral artery lesion is atherosis, which closely resembles atherosclerosis and links obstetrical syndromes to maternal vascular disease. Disorders of deep placentation associated with maternal vascular malperfusion are accompanied by characteristic alterations in angiogenic balance, and the ratio of placental growth factor to soluble fms-like tyrosine kinase-1 in the maternal circulation serves as a biomarker of this pathophysiologic process. Importantly, each obstetrical syndrome is associated with a stereotypic temporal pattern of angiogenic imbalance that reflects differences in disease burden, timing, and clinical expression. While substantial progress has been made in the prediction and prevention of preeclampsia, these concepts extend to other obstetrical syndromes, including fetal growth restriction, fetal death, and spontaneous preterm labor, supporting a unified biologic framework for early risk assessment and personalized prevention.

## 1. Introduction

The uniquely deep placentation in humans involves the physiological transformation of the decidual and inner third myometrial segments of the uterine spiral arteries to ensure adequate blood supply to the developing placenta and fetus and to support large brain development *in utero* (Brosens et al., 1967, Pijnenborg et al., 1983, Espinoza et al., 2006a, Allerkamp et al., 2021). Large encephalization has been a major invention of the human lineage, selected by environmental changes in Africa and permitted by the co-evolution of this uniquely deep placental invasion and its complex regulatory mechanisms at the maternal-fetal interface in the past two million years (Burton et al., 2015, Posta et al., 2024). The failure of this physiological transformation of the spiral arteries and the subsequent disrupted deep placentation was first detected in preeclampsia, a severe obstetrical syndrome only observed in humans (Brosens et al., 2011). Preeclampsia is a leading cause of maternal and perinatal morbidity and mortality, accounting for ~76, 000 maternal and ~500,000 fetal deaths peripartum annually around the globe (Erez et al., 2022, Jung et al., 2022). This can be attributed to gaps in our knowledge of the heterogeneous background of this syndrome and the consequent limitations of early diagnostic and therapeutic tools for its screening and effective, tailored prevention (Ness and Roberts, 1996, Hahn et al., 2015, Myatt and Roberts, 2015, Malone et al., 2022). Currently, preeclampsia is classified according to the severity of clinical symptoms and the gestational age at diagnosis or delivery (von Dadelszen et al., 2003). “Early-onset” preeclampsia that develops before the 34th week of gestation is more often associated with severe clinical presentation, fetal growth restriction (FGR), prematurity, as well as perinatal morbidity and mortality. It has a high incidence of placental histopathological abnormalities, mainly with maternal vascular malperfusion representing abnormal deep placentation, which is associated with excessive or atypical maternal immune response to the trophoblasts, placental oxidative stress and associated large transcriptomic changes, augmented placental production of anti-angiogenic factors, as well as consequent maternal systemic endothelial activation and inflammation; thus, it has been referred to as “placental preeclampsia”(Matthiesen et al., 2005, Varkonyi et al., 2011, Hansson et al., 2014, Redman et al., 2014, Saito and Nakashima, 2014, Blois et al., 2015, Szabo et al. 2015, Than et al., 2018, Rana et al., 2022, Robillard et al., 2022a, Mészáros et al., 2024). On the other hand, “late-onset” preeclampsia that develops after 34 weeks is more often associated with milder maternal symptoms and placental histologic features, and thus, it has often been referred as “maternal preeclampsia” (Robillard et al., 2022a). In spite of the milder and later symptoms in the majority of late-onset cases, it still poses a significant risk for maternal and fetal, acute (e.g., severe preeclampsia, HELLP syndrome, renal and liver disfunction) and chronic morbidities (e.g., cardiometabolic diseases, obesity, autoimmune diseases) in a large number of pregnancies (ACOG, 2002, Crispi et al., 2008, Soto et al., 2012, Redman et al., 2014, Verlohren et al., 2014, Scioscia et al., 2015, Robillard et al., 2019b, Birukov et al., 2020, Lekva et al., 2020, Roberts et al., 2021, Robillard et al., 2022b).

Deep placentation disorders reflected by maternal vascular malperfusion were found not only in preeclampsia but in all obstetrical syndromes to a varying extent (Kim et al., 2002, Kim et al., 2003, Ball et al., 2006). In addition, imbalances in maternal concentrations of circulating, placental-originated angiogenic (placental growth factor, PlGF) and anti-angiogenic (soluble fms-like tyrosine kinase-1, sFlt-1) factors were first reported in preeclampsia and then also in all other obstetrical syndromes. Subsequently, the anti-angiogenic imbalance in maternal circulation was found to be associated with maternal vascular malperfusion, reflecting deep placentation disorders (Kacerovsky et al., 2024). Since an abnormal PlGF/sFlt-1 ratio in midtrimester can identify women who will subsequently deliver placentas with maternal vascular malperfusion and develop placental-mediated obstetrical syndrome, it was suggested as a biomarker for the prediction of placental vascular disease (Korzeniewski et al., 2016).

The advances in omics technologies enabled the use of maternal blood molecular profiling as an ultimate non-invasive liquid biopsy of the health-status in pregnancy. With proteomics, four molecular subclasses of preeclampsia patients with distinct molecular pathologies were identified already in the first trimester (Than et al., 2022). The subsequent addition of placental histopathological examinations to longitudinal maternal liquid biopsy biomarker studies revealed the dynamic changes in preeclampsia molecular disease pathways throughout pregnancy, and identified common disease components with other obstetrical syndromes, including deep placentation disorders (Than et al., 2023, Than et al., 2024).

Clinical trials also unearthed effective preventive therapies (i.e., aspirin) for preterm preeclampsia patients, potentially those with deep placentation disorders, that are also effective in preventing a subgroup of patients developing other obstetrical syndromes, such as a subset of patients with FGR, fetal death, or spontaneous preterm birth (Bujold et al., 2010, Roberge et al., 2013, Hoffman et al., 2020, Kupka et al., 2023). Here, we summarize the literature on the shared placental disease pathways in these obstetrical syndromes and point out that the currently utilized diagnostic and therapeutic methods are probably not specific for preterm preeclampsia *per se* but for deep placentation disorders. This accumulating knowledge may be critical for the early and effective strategies that we can utilize for first trimester risk assessment and personalized prevention of the Great Obstetrical Syndromes with impaired deep placentation.

## 2. The great obstetrical syndromes

Obstetrical pathologies, including preeclampsia, spontaneous preterm labor/delivery with intact membranes, preterm prelabor rupture of the membranes (PPROM), FGR, and fetal death account for majority of adverse perinatal outcomes. These conditions are characterized by a long preclinical phase, fetal involvement, diverse clinical presentation, adaptive clinical manifestations, and gene environment interactions, and were coined by Roberto Romero as the ‘Great Obstetrical Syndromes’ (Di Renzo, 2009, Romero, 2009). Each syndrome is the clinical endpoint of different underlying pathomechanisms that may be chronic in nature and affect pregnancy from its early stages. Indeed, spontaneous preterm labor can result from intraamniotic infection that requires either delivery or antibiotic treatment or vascular placental disease that may benefit from aspirin prophylaxis. Therefore, different early pregnancy pathologies may lead to a similar clinical manifestation later in gestation (Romero, 2009). This may explain the failure of general preventive measures and the efficacy of prevention strategies based on specific mechanisms (i.e., vaginal progesterone to women with a short cervix). Thus, the syndromic nature of obstetrical diseases suggests that a battery of screening tests is required to identify patients at risk for obstetrical syndromes, the specific underlying mechanisms of disease (i. e., PlGF and sFlt-1 as markers for placental vascular disease), and the needed preventive or treatment strategy.

Obstetrical syndromes are adaptive in nature, meaning that they result from the maternal-fetal unit’s response to different pathological insults (Romero, 2009). High blood pressure in preeclampsia for instance is the maternal response to the abnormalities of the utero-placental vascular resistance, therefore, increased blood pressure is not the cause of preeclampsia but rather its main symptom, while FGR is the result of insufficient nutrition through the placenta, fetal infection or a genetic condition but not the primary pathology itself. By looking at these examples it becomes obvious why antihypertensive therapy alone fails to significantly reduce fetal and maternal morbidity and mortality in patients with preeclampsia.

## 3. Deep placentation disorders in the great obstetrical syndromes

Deep placentation involves a physiological transformation of the uterine spiral arteries to ensure adequate blood supply to the developing placenta and fetus (Fig. 1A). This is a unique vascular remodeling of the spiral arteries in the placental bed that undergo significant structural changes resulting from a deep extravillous trophoblast (EVT) invasion, replacing the musculo-elastic vessel walls by a mixture of fibrinoid material and fibrous tissue, gradually converting ~100–120 spiral arteries of the placental bed to large, tortuous channels (Brosens et al., 1967, Brosens et al., 2011). Initial alterations of the spiral arteries appear in the decidual segment as early as the 6th week and reach the inner third of the myometrial section of these arteries by the 20th week of gestation. The post-implantation phase of remodeling is dominated by uterine NK (uNK) cells whilst ongoing adaptation and transformation of the uterine arteries is orchestrated by separate and combined roles of uNKs and EVTs (Craven et al., 1998, Pállinger et al., 2023, Zhang et al., 2023). These physiological changes lead to an increased volume of intervillous blood flow that can be detected already by the 13th week (Pijnenborg et al., 1983, Espinoza et al., 2006a, Allerkamp et al., 2021, Surányi et al., 2023).

Parallel studies a decade ago also found that EVTs invade decidual veins and lymphatics starting from the 5–6th week of gestation, before EVTs start remodeling decidual spiral arteries (He et al., 2017, Windsperger et al., 2017, Moser et al., 2018). This was explained not to contribute to the materno-placental vascular connection directly, but to establish and/or expand the materno-fetal interface through an alternative vascular route. The significance of this finding in relevance to the topic addressed in this manuscript needs further investigation.

The failure of physiological transformation of the spiral arteries and a subsequent disrupted deep placentation is a shared pathological condition of all obstetrical syndromes (Brosens et al., 2011). Khong and Brosens studied the placental bed histological features in pregnancies complicated by preeclampsia and distinguished three types of defective deep placentation: (1) partial defective remodeling without obstructive vascular changes; (2) extensive defective remodeling without obstructive lesions; (3) extensive defective remodeling associated with obstructive vascular lesions (Khong and Brosens, 2011). The leading histological feature is the absence or partial transformation of the junctional zone of the myometrial spiral arteries, with or without obstructive lesions (e.g., thrombosis or atherosis), resulting in pathological villous pattern of maternal vascular malperfusion of the placenta (Redline et al., 2004, Staff et al., 2022, Brouwers et al., 2024). Early- and late-onset preeclampsia differ in the prevalence of maternal vascular malperfusion, the earlier the disease, the higher proportion and severity of vascular and villous maternal vascular malperfusion lesions (Moldenhauer et al., 2003, Nelson et al., 2014, Stanek, 2014) (Fig. 1B).

There are conflicting data regarding intraplacental and intervillous oxygen levels in deep placentation disorders, pointing to either hypoxia, hyperoxia, or ischemia-reperfusion injury (Burton, 2009, Burton et al., 2009a, Huppertz, 2023). From a histopathological point of view, the Amsterdam consensus classification system considers “accelerated villous maturation” and “distal villous hypoplasia” as pathological equivalences of maternal vascular malperfusion with a background of decidual vasculopathy, leading to decreased maternal blood flow (Redline et al., 2021). Of importance, this abnormal spiral artery remodeling and consequent defective deep placentation was also observed in a broader range of obstetrical syndromes including FGR, fetal death, preterm labor with intact membranes, PPROM, and placental abruption (Kim et al., 2002, Kim et al., 2003, Ball et al., 2006, Brosens et al., 2011, Khong and Brosens, 2011, Kim et al., 2015a, Kim et al., 2015b, Labarrere et al., 2017). The associated spiral artery atherosis in these obstetrical syndromes, an inflammatory lesion that contains lipid-laden and activated macrophages, has big resemblance to coronary artery atherosclerosis, suggesting a link between the Great Obstetrical Syndromes and their long-term cardiovascular sequelae (Labarrere et al., 2017, Staff et al., 2010, Staff et al., 2013, Staff and Redman, 2014, Jung et al., 2022). It is a big question why in certain women with deep placentation disorders preeclampsia will develop and in others not. It is probable that besides the extent of deep placentation disorders, certain maternal susceptibility factors also play a major role as suggested by the described shared and distinct risk factors between preeclampsia, SGA and FGR (Bartsch et al., 2016, Tsikouras et al., 2024).

### 3.1. Anti-angiogenic state in great obstetrical syndromes with deep placentation disorders

In normal pregnancies, pro-angiogenic factors [i.e., placental growth factor (PlGF) and vasculoendothelial growth factor (VEGF)] are essential for the development of placental and endothelial homeostasis. PlGF is detectable in maternal blood at early stages of pregnancy, and its concentrations gradually increase until peaking at ~33 weeks and then declines towards term (Levine et al., 2004, Romero et al., 2008). The concentrations of the anti-angiogenic factor sFlt-1 that inhibit the formation of blood vessels and the activity of angiogenic factors such as PlGF decrease in the course of pregnancies until ~30 weeks and increase thereafter until term (Levine et al., 2006, Erez et al., 2008, Romero et al., 2008). The ratio between angiogenic and anti-angiogenic factors represents the overall angiogenic balance that supports placental vessel formation and maternal vascular adaptation to pregnancy as well as systemic endothelial functions in the maternal circulation (Sundrani et al., 2013). The ratio between angiogenic and anti-angiogenic factors can be expressed in two forms: 1) the PlGF/sFlt-1 ratio (also coined as angiogenic index 1) is shifted upwards in the first two trimesters and then declines towards term (Romero et al., 2022) (Fig. 2); and 2) the sFlt-1/PlGF ratio (Maynard et al., 2003) that is currently being used in some of the risk assessment models for determining the risk for preeclampsia.

Defective angiogenesis has long been seen as a major cause of preeclampsia, and later an anti-angiogenic state was found to be key in the pathogenesis (Agarwal and Karumanchi, 2011, Jung et al., 2022). The placenta overexpresses the soluble, decoy form of the VEGF receptor (sFlt-1), leading to increased levels of sFlt-1 in the maternal circulation in women subsequently developing preeclampsia, in conjunction with the decreased maternal blood levels of free VEGF and PlGF, lowering angiogenic index 1 (Romero et al., 2022), or elevating the sFlt1/PlGF ratio as first reported by Maynard *et al*. (Maynard et al., 2003). In healthy pregnancies, sFlt-1 is released into the circulation by the placenta in decreasing and then stable amounts in the first and second trimesters (Erez et al., 2008), while in women with subsequent preeclampsia its levels start to increase 6–10 weeks prior to the onset of symptoms, with an earlier rise in earlier onset disease (Chaiworapongsa et al., 2005, Romero et al., 2008). Similarly, the anti-angiogenic soluble endoglin (sEng) - which suppresses autophagy in extravillous trophoblast, thereby reduces their invasion into the uterine myometrium and results in shallow placentation (Nakashima et al., 2013) - normally has decreasing levels from the first to the second trimester and then a gradual increase from 33 weeks onwards (Erez et al., 2008), while it has a more pronounced concentration elevation in women with subsequent preeclampsia, showing an earlier and steeper rise in cases of preterm compared to term disease (Thadhani et al., 2004, Levine et al., 2006, Erez et al., 2008, Romero et al., 2008, Kusanovic et al., 2009). In contrast, the decrease in PlGF levels is already detectable in the first trimester, especially in cases of early-onset preeclampsia, which continues throughout pregnancy (Thadhani et al., 2004, Romero et al., 2008, Verma et al., 2022). Those with preterm preeclampsia have a more pronounced anti-angiogenic state, starting earlier than those with term disease (Thadhani et al., 2004, Romero et al., 2008, Verma et al., 2022). Eclampsia is also associated with an anti-angiogenic state, similarly to severe, early-onset preeclampsia, suggesting a shared, common pathogenic pathway (Vaisbuch et al., 2011).

As evidence of causation, blood from women with preeclampsia demonstrated anti-angiogenic effects *in vitro*, which could be reversed by the addition of VEGF or PlGF (Ahmad and Ahmed, 2004). Moreover, the *in vivo* overexpression of sFlt-1 in pregnant animals induced preeclampsia-like symptoms including high blood pressure, proteinuria, systemic endothelial and related end-organ (e.g., kidney) damage which could be prevented with the addition of PlGF (Maynard et al., 2003, Szalai et al., 2014, Szalai et al., 2015). sEng, which was implicated in the neurological abnormalities, liver dysfunction and coagulopathies in preeclampsia (Venkatesha et al., 2006) was shown to reduce the vasodilating effect of TGF-β1 and cause increased capillary permeability in the kidneys, lungs and liver (Venkatesha et al., 2006).

An imbalance in the angiogenic/anti-angiogenic ratio is not specific to preeclampsia and was reported in mothers with SGA neonates (Chaiworapongsa et al., 2008, Erez et al., 2008, Romero et al., 2008, Chaiworapongsa et al., 2013, Chaiworapongsa et al., 2016), fetal death (Espinoza et al., 2007, Romero et al., 2010, Whitten et al., 2013), preterm labor with intact membranes (Chaiworapongsa et al., 2009), preterm PROM (Savasan et al., 2010), mirror syndrome (Espinoza et al., 2006b), and twin-to-twin transfusion syndrome (Kusanovic et al., 2008). Following the need to explain a common mechanism to these observations, Romero *et al*. further investigated the relation between placental lesions consistent with maternal vascular malperfusion and the anti-angiogenic state by measuring maternal blood angiogenic/anti-angiogenic factor concentrations in systematic cross-sectional and longitudinal studies involving women with preeclampsia and other obstetrical syndromes. The convincing evidence accumulated in these studies showed that sFlt-1, PlGF, and the sFlt/PlGF ratio have pathological changes in relation to maternal vascular malperfusion, and that this link is not only true for preeclampsia but also for all other obstetrical syndromes (Korzeniewski et al., 2016, Romero et al., 2022) (Fig. 3). Thus, angiogenic and anti-angiogenic factors should be considered as biomarkers of maternal vascular malperfusion rather than preeclampsia itself. Romero *et al*. also suggested the further subclassification of obstetrical syndromes based on the presence or absence of maternal vascular malperfusion to increase screening sensitivity and informativeness earlier in pregnancy. This was based on the findings that an angiogenic/anti-angiogenic imbalance is earlier and more pronounced in the presence of maternal vascular malperfusion compared to its absence in obstetrical syndromes. It was also concluded that a placental histology-based classification of these syndromes combined with accumulating information from omics studies will facilitate further implementation of placental biomarkers and improve their predictive performance in these disorders (Romero et al., 2022).

Indeed, our proteomics investigations in distinct ethnic populations using different proteomics platforms revealed four subclasses of preeclampsia patients, among whom early-onset placental disease and FGR were remarkable only in two subgroups (placental and metabolic subclasses), while the other two subclasses (anti-fetal rejection and extracellular matrix-related types) were mainly late-onset cases with underlying maternal disease and less placental and fetal pathologies (Than et al., 2023, Than et al., 2024). These findings were consistent with the separate placental and maternal origins of preeclampsia, the relation of deep placentation disorders with an anti-angiogenic state, and with a multi-stage pathological process originating from deep placentation disorders and resulting in an exaggerated pro-inflammatory state in preeclampsia patients (Roberts et al., 1989, Burton et al., 2009b, Burton et al., 2009c, Redman et al., 2014, Erez et al., 2023). Based on the maternal vascular malperfusion lesions and anti-angiogenic state detected in a portion of cases in all obstetrical syndromes, the above-described underlying mechanism may be active in patients with non-hypertensive clinical sequelae. The big question is why certain women will develop preeclampsia while others will develop other obstetrical syndromes (e.g., FGR without hypertension). This may be the result of an interplay of various maternal and paternal genetic, epigenetic as well as internal and external environmental factors, and it may also have evolutionary roots (Robillard et al., 2019a, Fülöp et al., 2025). There is also a growing body of evidence for cardiovascular predisposition in women developing preeclampsia (Perry et al., 2018).

## 4. Screening of great obstetrical syndromes with deep placentation disorders

Besides the extensive research focusing on the pathophysiology of preeclampsia, increasing efforts have been made to find predictive measures to identify women at an increased risk of developing this syndrome as early as possible. Initially, maternal history, clinical characteristics and biochemical markers used for screening of neural tube defects and aneuploidies (e.g., alpha-fetoprotein, beta-hCG, SP1, PAPP-A, inhibin A, uE3) were implicated in risk prediction in the first half of pregnancy (Moore and Redman, 1983, Than et al., 1993, Dekker, 1999), but these methods showed moderate value. Adding maternal blood or urine biochemical markers and biophysical parameters (e.g., Doppler ultrasonography indices) were hoped to increase screening performance, however, a systematic review conducted by WHO in 2004 still could not identify any clinically relevant predictors (Conde-Agudelo et al., 2004), mainly due to their low predictive value and specificity or the inconsistency of the data for the reliability of the markers studied (Sibai et al., 2005).

Later, the introduction of placental biomarkers (i.e., PlGF and PAPP-A) and multivariate screening algorithms radically increased the screening performance already in the first trimester over using maternal history alone as recommended by ACOG (ACOG, 2018) and NICE (NICE, 2019). Among several methods proposed, the Fetal Medicine Foundation’s first trimester preeclampsia screening algorithm appeared to be superior, widely used and validated by other research groups around the globe (Poon et al., 2009, Chaemsaithong et al., 2019, Zwertbroek et al., 2021, Rezende et al., 2024). The FMF first trimester algorithm combines maternal demographics, medical and obstetrical history, mean arterial pressure, as well as biomarkers of deep placentation (i.e., uterine artery pulsatility index (PI), maternal serum PlGF and PAPP-A). At a fixed false positive rate (FPR) of 10 %, the detection rate of this method reached 90 % for early-onset, 75 % for preterm, and 47 % for term preeclampsia (Chaemsaithong et al., 2022) (Fig. 4A), which prompted FIGO in 2019 to recommend offering first trimester screening of preeclampsia for all pregnant women based on this method (Poon et al., 2019). In light of the later revealed prevalence rate of deep placentation disorders in early-onset (75 %), preterm (55 %) and term (34 %) preeclampsia (Ogge et al., 2011) (Fig. 1B), the indirect evidence suggests that the detection rate of the FMF algorithm is primarily linked to deep placentation disorders in preeclampsia. This is further evidenced by the fact that the various algorithms developed by FMF for other obstetrical syndromes (i. e., SGA, FGR, fetal demise) are also built on these parameters, reflecting the early pathologic placentation events as underlying causes in many patients presenting later on with these syndromes (Papastefanou et al., 2020, Ashoor et al., 2022, Gérecz et al., 2025).

As preeclampsia pathogenesis progresses, and sFlt-1 levels rise in parallel with low PlGF, an anti-angiogenic shift - represented by the sFlt-1/PlGF ratio - in maternal circulation can be detected as early as 20–25 weeks of gestation in cases of early-onset preeclampsia (Kusanovic et al., 2009). As Romero *et al*. have shown, the sFlt-1/PlGF ratio becomes more elevated the closer we get to the development of preeclampsia (Romero et al., 2008). Soon thereafter, Verlohren et al. proposed the routine use of this ratio for the prediction of preeclampsia in the second half of pregnancy and for the risk stratification of women presenting at the clinic with clinical signs of preeclampsia (Verlohren et al., 2010). A ratio < 38 was considered reassuring, with only ~2 % of asymptomatic women developing preeclampsia later (Sovio et al., 2017). A ratio of 38–85 indicated moderate risk, requiring close monitoring, as time to delivery shortened significantly (Droge et al., 2021). A ratio ≥ 85 suggested high risk of short-term preeclampsia, often necessitating urgent care. In the INSPIRE trial, this ratio range had a 71.7 % positive predictive value to rule-in preeclampsia within the subsequent 4 weeks (Cerdeira et al., 2021), and in the ROPE study this ratio range had a 74 % positive predictive value for preeclampsia within 2 weeks if measured before 34 weeks (Rana et al., 2018). Besides the sFlt-1/PlGF ratio itself, Zeisler *et al*. also showed that the rate of change in the sFlt-1/PlGF ratio between two measurements is also of predictive value, therefore, a retest in 1–2 weeks following intermediate results is advisable (Zeisler et al., 2019). It was also suggested that in cases identified as high-risk in the first trimester, reassessing the sFlt-1/PlGF ratio every 4 weeks could also significantly reduce the rate of adverse pregnancy outcomes (Zeisler et al., 2019).

Regarding the anti-angiogenic state observed later in pregnancy in other obstetrical syndromes, the longitudinal study of Romero *et al*. has found a similar phenomenon in SGA to preeclampsia, however, changes in the maternal plasma concentrations of sEng, sFlt-1 and PlGF preceded the clinical presentation of preeclampsia, while only changes in sEng and PlGF were detected before the delivery of an SGA neonate (Romero et al., 2008). It was concluded that the differences in the profile of angiogenic/anti-angiogenic responses to intrauterine insults may determine whether a patient will deliver an SGA neonate, develop preeclampsia, or both. The degree of the severity of the angiogenic/anti-angiogenic imbalance is under debate and it seems that due to the heterogeneity of the underlying mechanisms leading to FGR, perturbations in this balance are related to the severity of the placental disease and correspond to Doppler abnormalities (Chaiworapongsa et al., 2008). The heterogeneity of underlying mechanisms leading to SGA was supported by a recent large epidemiology study by Robillard *et al*. that included 83,917 pregnancies (Robillard et al., 2024). It was found that 77 % of SGA cases were constitutional, while 23 % of SGA cases were FGR who had Doppler anomalies with underlying placental vascular disease. Of importance, only a quarter of these FGR cases had maternal disease (preeclampsia), while three quarters did not have.

Regarding the prediction of FGR, a recent meta-analysis reported that a > 33 sFlt-1/PlGF ratio measured in the late second or early third trimester had a sensitivity of 63 % and specificity of 84 % for the prediction of FGR. A cut-off of ≥ 85 improved sensitivity but reduced spec-ificity (Chen et al., 2022). Additionally, an observational study found an sFlt-1/PlGF ratio of > 38 detectable 4 weeks prior to delivery in 73 % of FGR cases without preeclampsia and in 100 % of FGR cases associated with preeclampsia (Herraiz et al., 2018). According to Gaccioli *et al.*, adding the sFlt-1/PlGF ratio to the measurement of the estimated fetal weight in the screening for delivery of an SGA neonate increases the positive likelihood ratio from 5.9 to 41.1 and the specificity from 92.2 % to 99.1 %, but resulted in the reduction of sensitivity from 46.2 % to 38.5 % (Gaccioli et al., 2018). On the other hand, Ciobanu *et al*. reported only marginal improvement in predictive performance when adding PlGF, sFlt-1, umbilical-, uterine- and middle cerebral artery PI to maternal factors and biometry (Ciobanu et al., 2019).

An abnormal angiogenic index-1 (PlGF/sFlt1 ratio) between 24 and 28 weeks of gestation can identify most women who will have fetal death (Chaiworapongsa et al., 2017). An angiogenic index-1 of < 2.5th centile had a positive likelihood ratio for predicting fetal death > 24 weeks of 14.6 and a relative risk of 29.1. Therefore, a maternal plasma angiogenic index-1 value of < 2.5th centile at 24–28 weeks of gestation carries a 29-fold increased risk of subsequent fetal death and identifies 55 % of subsequent fetal deaths with a false-positive rate of 3.5 %.

Multiple studies have also evaluated the relation between the levels of angiogenic and anti-angiogenic factors and the rate of preterm delivery. Salahuddin *et al*. reported an increased sFlt-1/PlGF ratio in women with adverse pregnancy outcomes including preterm delivery and found an elevation in sFlt-1/PlGF ratio inversely related to the time until delivery (Salahuddin et al., 2016). Rana *et al*. reported an 86 % delivery rate within 2 weeks with an sFlt-1/PlGF ratio of ≥ 85 compared to only 15.8 % when this was < 85 in women at < 34 weeks of gestation with the clinical suspicion of early-onset preeclampsia (Rana et al., 2012). In pregnancies complicated by preeclampsia before 35 weeks, a PlGF level of < 100 pg/ml predicted preterm delivery with a sensitivity of 81.7 % and a specificity of 85.3 % (Barton et al., 2020). The association between angiogenic factors and the rate of stillbirth has also been examined but remains unclear. Overall, this evidence suggests that angiogenic and anti-angiogenic factors and their ratio are good biomarkers for placental vascular disease, and they can potentially be useful in predicting adverse pregnancy outcomes related to defective deep placentation.

## 5. Prevention of great obstetrical syndromes with deep placentation disorders

The urge to find effective screening algorithms for the identification of women at increased risk of deep placentation disorders was also facilitated by multiple studies suggesting the usefulness of aspirin therapy in the prevention of preeclampsia. This positive effect of aspirin was first reported in smaller studies by Goodlin *et al*. in 1978 (Goodlin et al., 1978) and then Beaufils *et al*. (Beaufils et al., 1985). Since then these were followed by numerous randomized clinical trials examining the potential use of aspirin (Caritis et al., 1998, Villa et al., 2013, Odibo et al., 2015, Rolnik et al., 2017, Lin et al., 2022, Sinha et al., 2023), however, for more than three decades the results were inconsistent due to the differences in the dose of aspirin used, the timing of the initiation of treatment, the size of the study populations, and the patient selection criteria (Askie et al., 2007). Early initiation of aspirin treatment, however, was suspected to decrease the disease prevalence, and it was the meta-analysis from Bujold *et al*. which first highlighted the importance of starting aspirin < 16 weeks in high-risk patients, as it resulted in a reduction of preeclampsia risk by 50 % versus 20 % if the treatment started only later in gestation (Bujold et al., 2014). Controversy has also existed regarding the dosage of aspirin to be used. The team of Bujold *et al*. (Roberge et al., 2017) reported that among those who were treated with aspirin < 16 weeks of gestation, the risk-reduction effect was directly related to the dose of aspirin administered. On the other hand, both meta-analyses confirmed that aspirin started > 16 weeks had only modest or no effect irrespective of the dose administered. These results support the notion that low-dose aspirin administration has a supportive effect on deep placentation and its administration needs to begin before the completion of trophoblast invasion. A subsequent meta-analysis by Roberge *et al*. suggested that aspirin should be administered at a minimum dose of 100 mg to reach its potential preventive effect (Roberge et al., 2017), and later a dose of 150–162 mg was recommended to have an optimal antiplatelet effect (Ghesquiere et al., 2023). In addition to these, the ASPRE randomized clinical trial by Rolnik *et al*. confirmed the usefulness of aspirin and determined that its preventive effect was more pronounced in cases of preterm rather than term preeclampsia (Fig. 4B) (Rolnik et al., 2017). The study population included 26,941 patients who were screened with the FMF algorithm and amongst them 2971 had high-risk screening results. Altogether 1620 women were included in the prevention study, 798 in the aspirin treatment and 822 in the placebo arm. Aspirin treatment reduced the risk for early-onset preeclampsia by 82 %, for preterm preeclampsia by 62 % and by term preeclampsia by 5 %.

As summarized here, deep placentation disorders can lead to the development not only of preeclampsia, but other obstetrical syndromes, as well. Based on this common pathological background, the effect of aspirin had been investigated in relation to other obstetrical syndromes. The ASPIRIN randomized clinical trial by Hoffman *et al*. included 11,976 nulliparous women with a singleton pregnancy and randomly assigned them to receive low-dose (81 mg daily) aspirin (n = 5990) or placebo (n = 5986). It showed the risk reduction in the aspirin arm for preterm birth < 37 weeks by 11 %, for early preterm delivery (<34 weeks) by 25 %, for perinatal mortality by 14 %, for fetal loss by 14 %, and for hypertensive disorders of pregnancy < 34 weeks by 62 % while other adverse maternal and neonatal outcomes were similar in the two groups (Hoffman et al., 2020). The meta-analyses by Bujold *et al*. showed that aspirin started < 16 weeks also reduced the risk of FGR by 56 % and preterm birth by 78 % besides reducing gestational hypertension risk by 38 % (Bujold et al., 2010, Bujold et al., 2014) (Fig. 4C). Beyond these, the meta-analysis of Roberge *et al*. also revealed the preventive effect of aspirin for perinatal death, especially when started < 16 weeks by 59 % (Roberge et al., 2013). The risk of placental abruption was not significantly reduced by aspirin intake, however, there may be a positive effect if administered < 16 weeks (Roberge et al., 2018). Aspirin may also have a positive effect on the rate of recurrent preterm birth in case of women with history of preterm birth as shown by Kupka et al. in a Swedish register-based cohort study (Kupka et al., 2023). Of interest, a recent meta-analysis found pravastatin to also have risk-reducing effects for the development of preeclampsia, FGR, preterm birth, and neonatal NICU admissions, further strengthening the evidence on the common pathophysiological roots of the Great Obstetrical Syndromes (Mészáros et al., 2022).

## 6. Summary and conclusions

Accumulating evidence shows that the impairment of deep placentation, originally thought to be specific for preeclampsia, is present as a common pathomechanism in varying extent in all obstetrical syndromes. This is associated with an anti-angiogenic state developing in the maternal circulation, which gives us the opportunity for a timely diagnosis. Clinical trials have also proven that aspirin therapy, if started < 16 weeks, is a very effective means of prevention for these placentation disorders in a yet not fully explored manner. High-dimensional studies have also demonstrated other molecular disease pathways activated in other subsets of patients with preeclampsia and other obstetrical syndromes. The major aims should now be to (1) develop reliable diagnostic and preventive methods for these additional molecular pathways; (2) reveal why the same or overlapping disease pathways lead to the development of clinically distinct diseases (e.g., preeclampsia associated with FGR vs. FGR; and (3) develop a more detailed taxonomy of these syndromes based on not only their clinical but also placental and molecular characteristics. This would lay the ground for the early and effective strategies for risk assessment and personalized prevention of the Great Obstetrical Syndromes.

## Figures and Tables

**Fig. 1.**
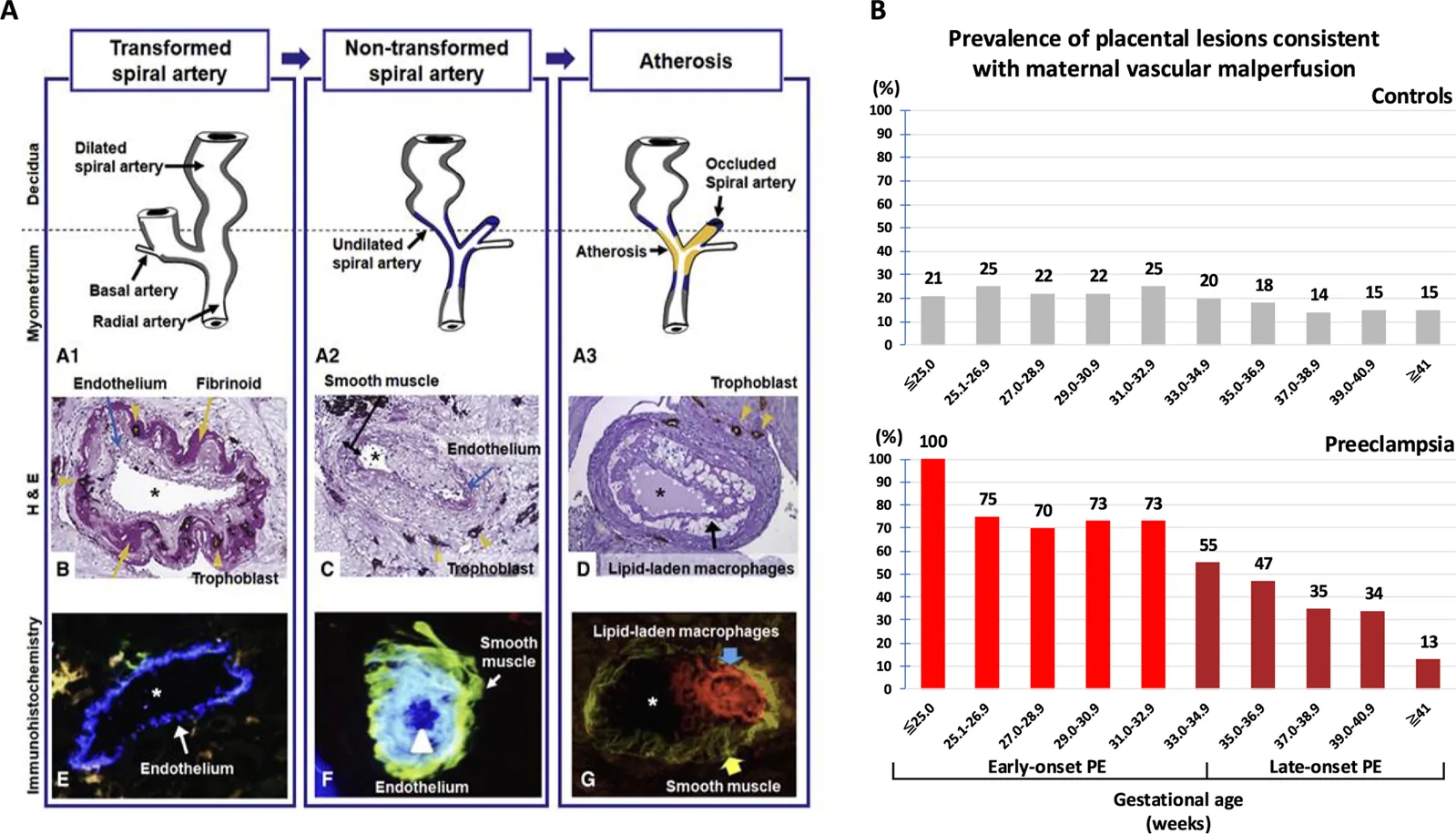
Normal and defective spiral artery transformation. Left panel (A): During normal placentation, spiral arteries undergo physiological changes, while in preeclampsia the transformation of the myometrial segment of the spiral arteries is defective **(A1–3)**. Non-transformed spiral arteries often show signs of atherosis, characterized by the presence of lipid-filled macrophages within the lumen. Hematoxylin-eosin stains: the presence of intramural trophoblasts (*arrowheads*) and fibrinoid degeneration (*arrows*) can be seen in normal pregnancy specimens **(B)**, while non-transformed spiral arteries retain smooth muscle, lack the presence of trophoblasts **(C)** or contain lipid-filled macrophages in acute atherotic lesions (*arrows*) **(D)**. Immunostaining: presence of endothelium (*blue*) in vessels with normal trophoblastic invasion **(E)**, non-transformed spiral artery with smooth muscle cells (*green, arrow*) and endothelium (*blue, arrowhead*) **(F)**, or atherotic lesions with numerous macrophages (*red, blue arrow*) and smooth muscle cells in the vessel wall (*green, yellow arrow*) **(G)**. Images were taken from the central part of the basal plate of the placenta (Espinoza et al. 2006a, Labarrere et al. 2017). A triple-antibody technique with antibodies to α-smooth muscle actin to identify vascular smooth muscle cells, cytokeratin to identify extravillous trophoblasts and intra-arterial trophoblasts, and von Willebrand factor to identify endothelial cells was used to detect complete or partial failure of physiologic transformation and physiologic transformation of spiral arteries (Labarrere et al. 2017). The figure was adapted with permission from Jung *et al*. (Jung et al. 2022). ***Right panel (B):*** The prevalence of placental lesions consistent with maternal vascular malperfusion is rather low and constant according to gestational age at delivery in non-hypertensive (control) pregnancies. However, in pregnancies complicated with preeclampsia, the prevalence of maternal vascular malperfusion lesions is high in early-onset cases with a decreasing trend towards term. Data was adapted with permission from Ogge *et al*. (Ogge et al. 2011).

**Fig. 2.**
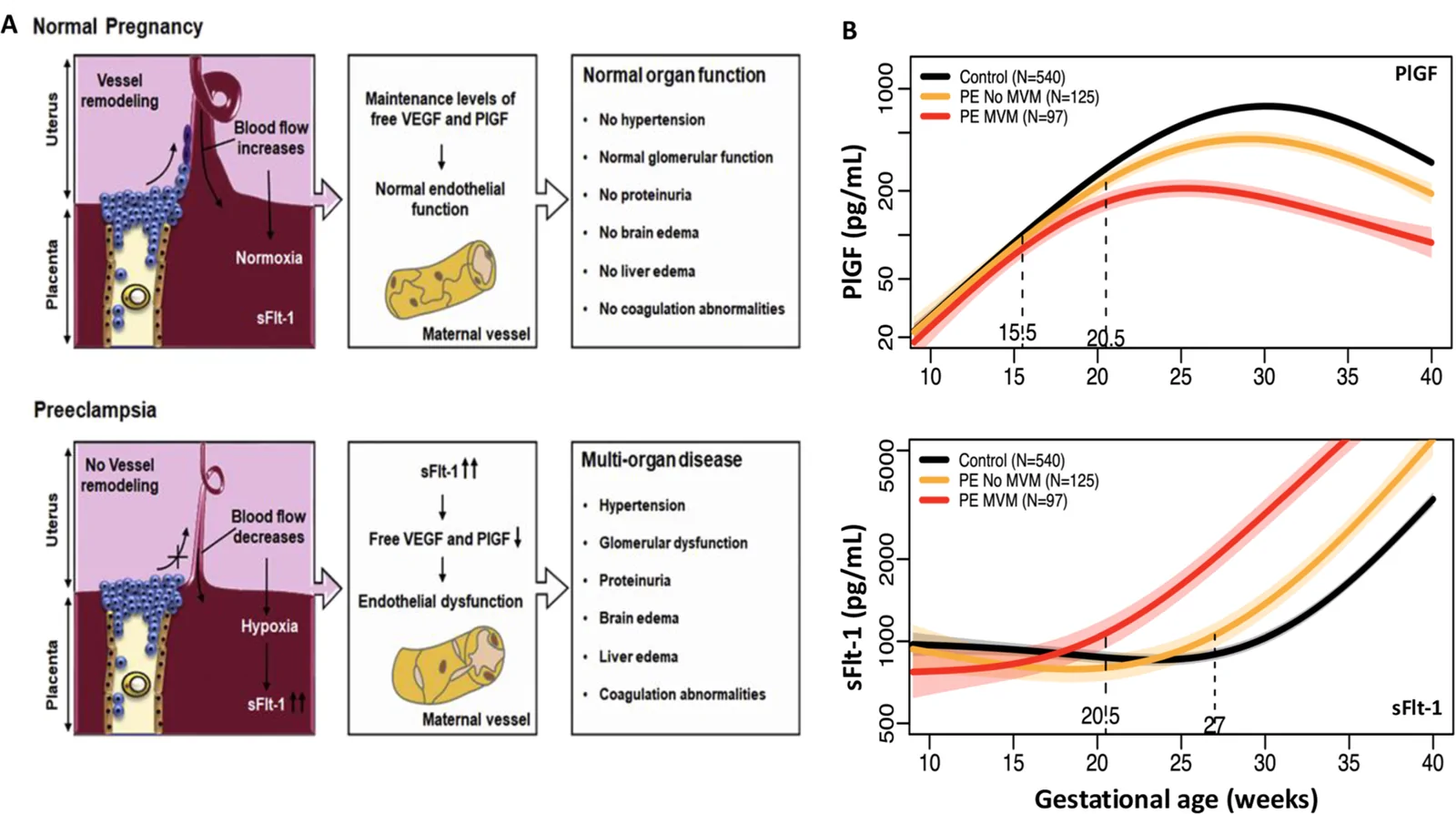
Spiral artery remodeling and the angiogenic / anti-angiogenic (im)balance. In normal pregnancies, the remodeling of the spiral arteries increases placental blood flow and maintains a normal angiogenic/anti-angiogenic balance with high PlGF and low sFlt-1 maternal plasma levels till 30 weeks of gestation **(A – top panel, B)**. In preeclampsia with maternal vascular malperfusion (MVM), defective spiral artery remodeling results in the placental overexpression of sFlt-1 and the sequestration of PlGF in the maternal circulation, an anti-angiogenic state, leading to endothelial dysfunction and consequent hypertension, proteinuria and multiorgan failure (**A – bottom panel, B)**. Figures were adapted with permission from Erez *et al*. (Erez et al. 2022) and Romero *et al*. (Romero et al. 2022).

**Fig. 3.**
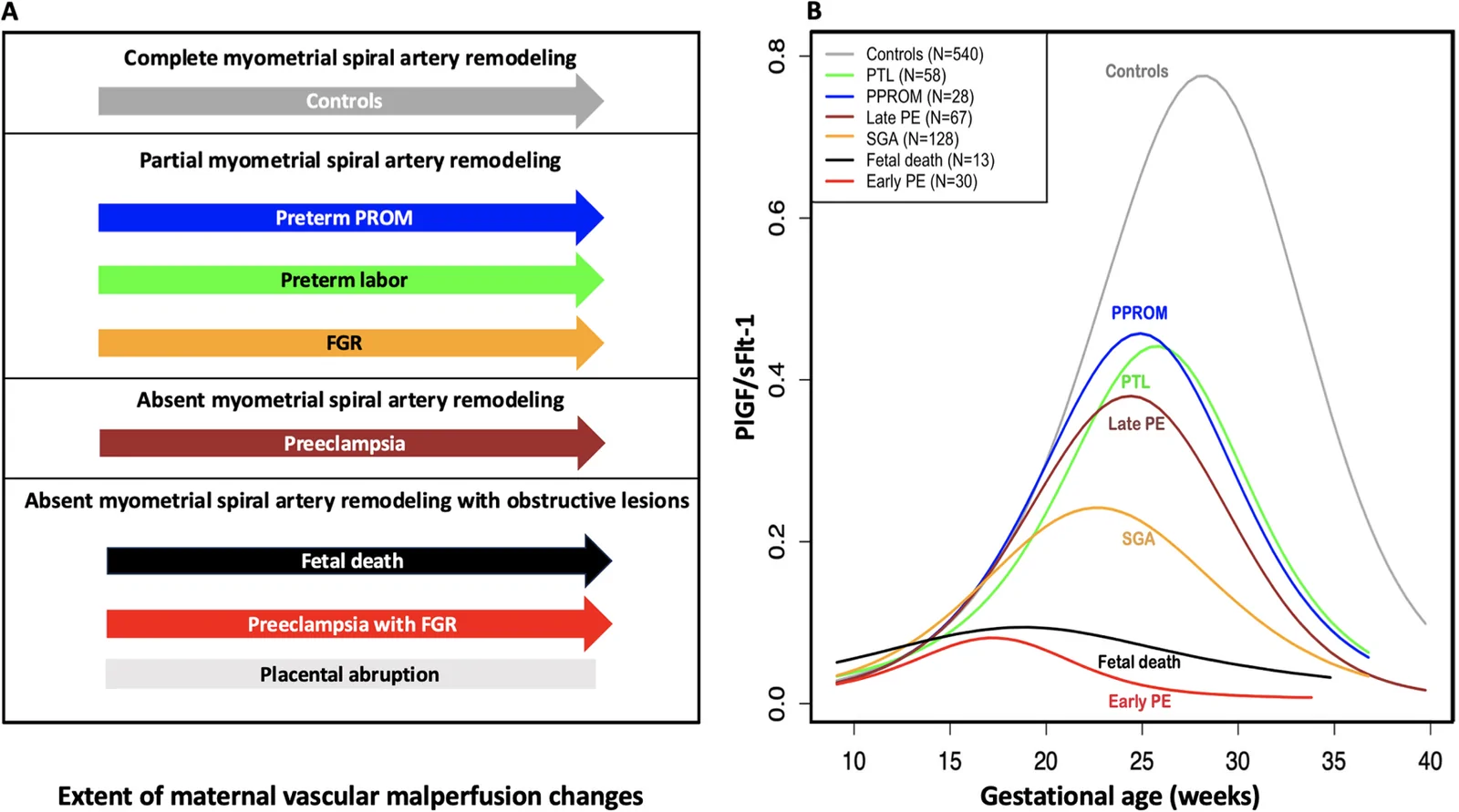
Associations between deep placentation disorders, anti-angiogenic state and adverse pregnancy outcomes. **(A)** The extent of defective deep placentation is larger in obstetrical syndromes with greater adverse pregnancy outcomes. **(B)** The decrease in maternal plasma PlGF/sFlt-1 ratio (angiogenic index 1), suggestive of an antiangiogenic state, is more extensive in a subset of patients with obstetrical syndromes associated with maternal vascular malperfusion and greater adverse pregnancy outcomes. The color of arrows in the left panel corresponds to the color of curves in the right panel. (a) The figure was adapted with permission from Brosens et al. (2011) **(A)**. (b) The figure was adapted with permission from Romero et al. (2022) **(B)**.

**Fig. 4.**
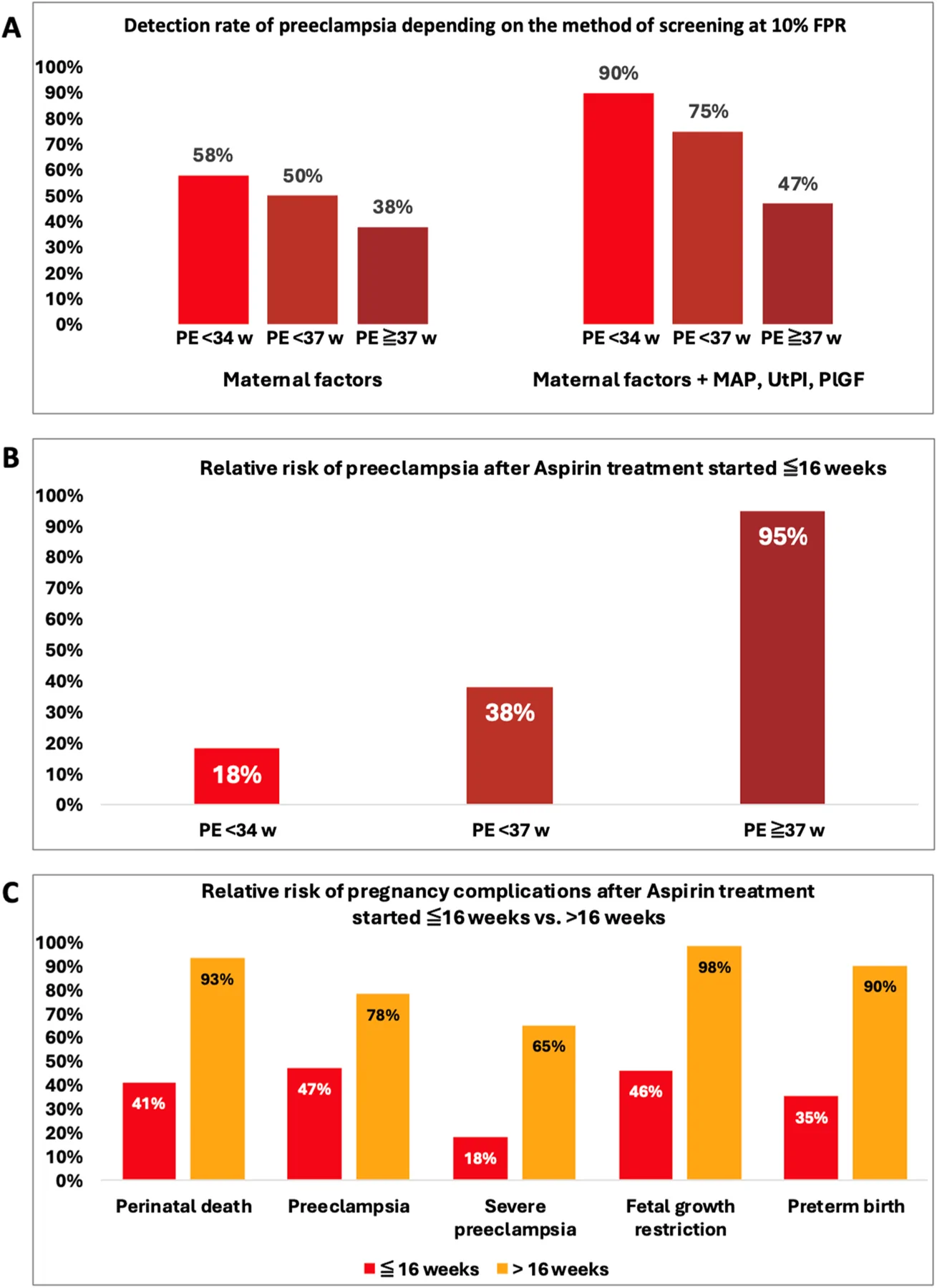
Screening and aspirin prevention of deep placentation disorder in preeclampsia and other obstetrical syndromes. The first trimester detection rate of early-onset, preterm and term preeclampsia is significantly increased if mean arterial pressure as well as deep placentation biomarkers (i.e., uterine artery pulsatility index and PlGF) are used in addition to maternal factors. The reduction rate of early-onset, preterm and term preeclampsia development is significantly higher if prevention with aspirin is started before 16 weeks of gestation. The reduction rate of adverse outcomes related to abnormal placentation in various obstetrical syndromes is significantly higher if prevention with aspirin is started < 16 weeks of gestation. (a) Data was adapted with permission from the Fetal Medicine Foundation (https://courses.fetalmedicine.com/) **(A)**. (b) Data was adapted with permission from Rolnik *et al*. (Rolnik et al. 2017) **(B)**. (c) Data was adapted with permission from Bujold *et al*. (Bujold et al. 2014) **(C)**.

## References

[R1] ACOG, 2002. ACOG committee on practice bulletins, ACOG practice bulletin No. 33: diagnosis and management of preeclampsia and eclampsia. Obstet. Gynecol 99, 159–167. 10.1016/s0029-7844(01)01747-1 16175681

[R2] ACOG, 2018. ACOG committee opinion No. 743 summary: low-dose aspirin use during pregnancy. Obstet. Gynecol 132, 254–256. 10.1097/AOG.0000000000002709 29939936

[R3] Agarwal I, Karumanchi SA, 2011. Preeclampsia and the anti-angiogenic state. Pregnancy Hypertens. 1, 17–21. 10.1016/j.preghy.2010.10.007 PMC3087187 21709826

[R4] Ahmad S, Ahmed A, 2004. Elevated placental soluble vascular endothelial growth factor receptor-1 inhibits angiogenesis in preeclampsia. Circ. Res 95, 884–891. 10.1161/01.RES.0000147365.86159.f5 15472115

[R5] Allerkamp HH, , 2021. Something old, something new: digital quantification of uterine vascular remodelling and trophoblast plugging in historical collections provides new insight into adaptation of the utero-placental circulation. Hum. Reprod 36, 571–586. 10.1093/humrep/deaa303 33600565

[R6] Ashoor G, , 2022. Development and validation of model for prediction of placental dysfunction-related stillbirth from maternal factors, fetal weight and uterine artery Doppler at mid-gestation. Ultrasound Obstet. Gynecol 59, 61–68. 10.1002/uog.24795 34643306

[R7] Askie LM, , 2007. Antiplatelet agents for prevention of pre-eclampsia: a meta-analysis of individual patient data. Lancet 369, 1791–1798. 10.1016/S0140-6736(07)60712-0 17512048

[R8] Ball E, , 2006. Late sporadic miscarriage is associated with abnormalities in spiral artery transformation and trophoblast invasion. J. Pathol 208, 535–542. 10.1002/path.1927 16402350

[R9] Barton JR, , 2020. Placental growth factor predicts time to delivery in women with signs or symptoms of early preterm preeclampsia: a prospective multicenter study. Am. J. Obstet. Gynecol 222, 259 e1–259 e11. 10.1016/j.ajog.2019.09.003 31518550

[R10] Bartsch E, , 2016. Clinical risk factors for pre-eclampsia determined in early pregnancy: systematic review and meta-analysis of large cohort studies. BMJ 353, i1753. 10.1136/bmj.i1753 PMC4837230 27094586

[R11] Beaufils M, , 1985. Prevention of pre-eclampsia by early antiplatelet therapy. Lancet 1, 840–842. 10.1016/s0140-6736(85)92207-x 2858710

[R12] Birukov A, , 2020. Myocardial evaluation of post-preeclamptic women by CMR: is early risk stratification possible. JACC Cardiovasc. Imaging 13, 1291–1293. 10.1016/j.jcmg.2020.01.005 32061559

[R13] Blois SM, , 2015. A potential pathophysiological role for galectins and the reninangiotensin system in preeclampsia. Cell Mol. Life Sci 72, 39–50. 10.1007/s00018-014-1713-1 PMC11113509 25192660

[R14] Brosens I, , 1967. The physiological response of the vessels of the placental bed to normal pregnancy. J. Pathol. Bacteriol 93, 569–579. 10.1002/path.1700930218 6054057

[R15] Brosens I, , 2011. The “Great Obstetrical Syndromes” are associated with disorders of deep placentation. Am. J. Obstet. Gynecol 204, 193–201. 10.1016/j.ajog.2010.08.009 PMC3369813 21094932

[R16] Brouwers L, , 2024. Prevalence of placental bed spiral artery pathology in preeclampsia and fetal growth restriction: a prospective cohort study. Placenta 156, 1–9. 10.1016/j.placenta.2024.08.010 39214009

[R17] Bujold E, , 2010. Prevention of preeclampsia and intrauterine growth restriction with aspirin started in early pregnancy: a meta-analysis. Obstet. Gynecol 116, 402–414. 10.1097/AOG.0b013e3181e9322a 20664402

[R18] Bujold E, , 2014. Low-dose aspirin for prevention of adverse outcomes related to abnormal placentation. Prenat. Diagn 34, 642–648. 10.1002/pd.4403 24799357

[R19] Burton GJ, 2009. Oxygen, the Janus gas; its effects on human placental development and function. J. Anat 215, 27–35. 10.1111/j.1469-7580.2008.00978.x PMC2714636 19175804

[R20] Burton GJ, , 2009c. Placental endoplasmic reticulum stress and oxidative stress in the pathophysiology of unexplained intrauterine growth restriction and early onset preeclampsia. Placenta 30 (A), S43–S48. 10.1016/j.placenta.2008.11.003 PMC2684656 19081132

[R21] Burton GJ, , 2009a. Regulation of vascular growth and function in the human placenta. Reproduction 138, 895–902. 10.1530/REP-09-0092 19470597

[R22] Burton GJ, , 2009b. Rheological and physiological consequences of conversion of the maternal spiral arteries for uteroplacental blood flow during human pregnancy. Placenta 30, 473–482. 10.1016/j.placenta.2009.02.009 PMC2697319 19375795

[R23] Burton GJ, , 2015. Human evolution: brain, birthweight and the immune system. Philos. Trans. R. Soc. Lond. B Biol. Sci 370, 20140061. 10.1098/rstb.2014.0061 PMC4305162 25602065

[R24] Caritis S, 1998. Low-dose aspirin to prevent preeclampsia in women at high risk. National Institute of Child Health and Human Development Network of Maternal-Fetal Medicine Units. N Engl J Med 338, 701–705. 10.1056/NEJM199803123381101. 9494145

[R25] Cerdeira AS, , 2021. Performance of soluble fms-like tyrosine kinase-1-to-placental growth factor ratio of >/=85 for ruling in preeclampsia within 4 weeks. Am. J. Obstet. Gynecol 224, 322–323. 10.1016/j.ajog.2020.11.007 33207235

[R26] Chaemsaithong P, , 2019. Prospective evaluation of screening performance of firsttrimester prediction models for preterm preeclampsia in an Asian population. Am. J. Obstet. Gynecol 221, 650.e1–650.e16. 10.1016/j.ajog.2019.09.041 31589866

[R27] Chaemsaithong P, , 2022. First trimester preeclampsia screening and prediction. Am. J. Obstet. Gynecol 226, S1071–S1097.e2. 10.1016/j.ajog.2020.07.020 32682859

[R28] Chaiworapongsa T, , 2005. Plasma soluble vascular endothelial growth factor receptor-1 concentration is elevated prior to the clinical diagnosis of pre-eclampsia. J. Matern. Fetal Neonatal Med 17, 3–18. 10.1080/14767050400028816 15804781

[R29] Chaiworapongsa T, , 2008. The maternal plasma soluble vascular endothelial growth factor receptor-1 concentration is elevated in SGA and the magnitude of the increase relates to Doppler abnormalities in the maternal and fetal circulation. J. Matern. Fetal Neonatal Med 21, 25–40. 10.1080/14767050701832833 PMC7062304 18175242

[R30] Chaiworapongsa T, , 2009. A subset of patients destined to develop spontaneous preterm labor has an abnormal angiogenic/anti-angiogenic profile in maternal plasma: evidence in support of pathophysiologic heterogeneity of preterm labor derived from a longitudinal study. J. Matern. Fetal Neonatal Med 22, 1122–1139. 10.3109/14767050902994838 PMC3437777 19916710

[R31] Chaiworapongsa T, , 2013. Maternal plasma concentrations of angiogenic/antiangiogenic factors in the third trimester of pregnancy to identify the patient at risk for stillbirth at or near term and severe late preeclampsia. Am. J. Obstet. Gynecol 208, 287e1–287e15. 10.1016/j.ajog.2013.01.016 PMC4086897 23333542

[R32] Chaiworapongsa T, , 2016. The use of angiogenic biomarkers in maternal blood to identify which SGA fetuses will require a preterm delivery and mothers who will develop pre-eclampsia. J. Matern. Fetal Neonatal Med 29, 1214–1228. 10.3109/14767058.2015.1048431 PMC5769689 26303962

[R33] Chaiworapongsa T, , 2017. The prediction of fetal death with a simple maternal blood test at 20–24 weeks: a role for angiogenic index-1 (PlGF/sVEGFR-1 ratio). Am. J. Obstet. Gynecol 217, 682.e1–682.e13. 10.1016/j.ajog.2017.10.001 PMC5951183 29037482

[R34] Chen W, , 2022. Diagnostic capacity of sFlt-1/PlGF ratio in fetal growth restriction: A systematic review and meta-analysis. Placenta 127, 37–42. 10.1016/j.placenta.2022.07.020 35952596

[R35] Ciobanu A, , 2019. Prediction of small for gestational age neonates: screening by maternal factors, fetal biometry, and biomarkers at 35–37 weeks' gestation. Am. J. Obstet. Gynecol 220, 486.e1–486.e11. 10.1016/j.ajog.2019.01.227 30707967

[R36] Conde-Agudelo A, , 2004. World Health Organization systematic review of screening tests for preeclampsia. Obstet. Gynecol 104, 1367–1391. 10.1097/01.AOG.0000147599.47713.5d 15572504

[R37] Craven CM, , 1998. Decidual spiral artery remodelling begins before cellular interaction with cytotrophoblasts. Placenta 19, 241–252. 10.1016/s0143-4004(98)90055-8 9639319

[R38] Crispi F, , 2008. Predictive value of angiogenic factors and uterine artery Doppler for early- versus late-onset pre-eclampsia and intrauterine growth restriction. Ultrasound Obstet. Gynecol 31, 303–309. 10.1002/uog.5184 18058842

[R39] von Dadelszen P, , 2003. Subclassification of preeclampsia. Hypertens. Pregnancy 22, 143–148. 10.1081/PRG-120021060 12908998

[R40] Dekker GA, 1999. Risk factors for preeclampsia. Clin. Obstet. Gynecol 42, 422–435. 10.1097/00003081-199909000-00002 10451762

[R41] Di Renzo GC, 2009. The great obstetrical syndromes. J. Matern. Fetal Neonatal Med 22, 633–635. 10.1080/14767050902866804 19736613

[R42] Droge LA, , 2021. Prediction of preeclampsia-related adverse outcomes with the sFlt-1 (Soluble fms-Like Tyrosine Kinase 1)/PlGF (Placental Growth Factor)-ratio in the clinical routine: a real-world study. Hypertension 77, 461–471. 10.1161/HYPERTENSIONAHA.120.15146 33280406

[R43] Erez O, , 2008. The change in concentrations of angiogenic and anti-angiogenic factors in maternal plasma between the first and second trimesters in risk assessment for the subsequent development of preeclampsia and small-for-gestational age. J. Matern. Fetal Neonatal Med 21, 279–287. 10.1080/14767050802034545 PMC2846114 18446652

[R44] Erez O, , 2022. Preeclampsia and eclampsia: the conceptual evolution of a syndrome. Am. J. Obstet. Gynecol 226, S786–S803. 10.1016/j.ajog.2021.12.001 PMC8941666 35177220

[R45] Erez O, , 2023. Perturbations in kinetics of the thrombin generation assay identify women at risk of preeclampsia in the first trimester and provide the rationale for a preventive approach. Am. J. Obstet. Gynecol 228, 580.e1–580.e17. 10.1016/j.ajog.2022.11.1276 PMC10149548 36368431

[R46] Espinoza J, , 2006b. A role of the anti-angiogenic factor sVEGFR-1 in the 'mirror syndrome' (Ballantyne's syndrome). J. Matern. Fetal Neonatal Med 19, 607–613. 10.1080/14767050600922677 PMC6941849 17118734

[R47] Espinoza J, , 2006a. Normal and abnormal transformation of the spiral arteries during pregnancy. J. Perinat. Med 34, 447–458. 10.1515/JPM.2006.089 PMC7062302 17140293

[R48] Espinoza J, , 2007. Unexplained fetal death: another anti-angiogenic state. J. Matern. Fetal Neonatal Med 20, 495–507. 10.1080/14767050701413022 PMC7062303 17674262

[R49] Fülöp V, , 2025. The effects of environmental factors on the embryonic and late postembryonic life through epigenetic mechanisms. Orv. Hetil 166, 799–807. 10.1556/650.2025.33305 40418583

[R50] Gaccioli F, , 2018. Screening for fetal growth restriction using ultrasound and the sFLT1/PlGF ratio in nulliparous women: a prospective cohort study. Lancet Child Adolesc. Health 2, 569–581. 10.1016/S2352-4642(18)30129-9 PMC6473551 30119716

[R51] Gérecz B, , 2025. Fetal growth restriction as the disease of the placenta. Orv. Hetil 166, 331–340. 10.1556/650.2025.33254 40023836

[R52] Ghesquiere L, , 2023. Comparing aspirin 75–81 mg vs 150–162 mg for prevention of preterm preeclampsia: systematic review and meta-analysis. Am. J. Obstet. Gynecol. MFM 5, 101000. 10.1016/j.ajogmf.2023.101000 37146687

[R53] Goodlin RC, , 1978. Aspirin for the treatment of recurrent toxaemia. Lancet 2, 51. 10.1016/s0140-6736(78)91367-3 78253

[R54] Hahn S, , 2015. Biomarker development for presymptomatic molecular diagnosis of preeclampsia: feasible, useful or even unnecessary? Expert Rev. Mol. Diagn 15, 617–629. 10.1586/14737159.2015.1025757 PMC4673513 25774007

[R55] Hansson SR, , 2014. Oxidative stress in preeclampsia and the role of free fetal hemoglobin. Front. Physiol 5, 516. 10.3389/fphys.2014.00516 PMC4292435 25628568

[R56] He N, , 2017. Human extravillous trophoblasts penetrate decidual veins and lymphatics before remodeling spiral arteries during early pregnancy. PLOS One 12, e0169849. 10.1371/journal.pone.0169849 PMC5230788 28081266

[R57] Herraiz I, , 2018. Longitudinal change of sFlt-1/PlGF ratio in singleton pregnancy with early-onset fetal growth restriction. Ultrasound Obstet. Gynecol 52, 631–638. 10.1002/uog.18894 28876491

[R58] Hoffman MK, , 2020. Low-dose aspirin for the prevention of preterm delivery in nulliparous women with a singleton pregnancy (ASPIRIN): a randomised, double-blind, placebo-controlled trial. Lancet 395, 285–293. 10.1016/S0140-6736(19)32973-3 PMC7168353 31982074

[R59] Huppertz B, 2023. Placental physioxia is based on spatial and temporal variations of placental oxygenation throughout pregnancy. J. Reprod. Immunol 158, 103985. 10.1016/j.jri.2023.103985 37406413

[R60] Jung E, , 2022. The etiology of preeclampsia. Am. J. Obstet. Gynecol 226, S844–S866. 10.1016/j.ajog.2021.11.1356 PMC8988238 35177222

[R61] Kacerovsky M, , 2024. Predictive value of the sFlt-1/PlGF ratio and interleukin-6 for the presence of placental lesions in spontaneous preterm labor with intact membranes with delivery within 7 days. Fetal Diagn. Ther 51, 539–549. 10.1159/000540203 PMC11633894 38964308

[R62] Khong Y, Brosens I, 2011. Defective deep placentation. Best. Pract. Res. Clin. Obstet. Gynaecol 25, 301–311. 10.1016/j.bpobgyn.2010.10.012 21109492

[R63] Kim YM, , 2002. Failure of physiologic transformation of the spiral arteries in the placental bed in preterm premature rupture of membranes. Am. J. Obstet. Gynecol 187, 1137–1142. 10.1067/mob.2002.127720 12439491

[R64] Kim YM, , 2003. Failure of physiologic transformation of the spiral arteries in patients with preterm labor and intact membranes. Am. J. Obstet. Gynecol 189, 1063–1069. 10.1067/s0002-9378(03)00838-x 14586356

[R65] Kim YM, , 2015a. The frequency of acute atherosis in normal pregnancy and preterm labor, preeclampsia, small-for-gestational age, fetal death and midtrimester spontaneous abortion. J. Matern. Fetal Neonatal Med 28, 2001–2009. 10.3109/14767058.2014.976198 PMC4427552 25308204

[R66] Kim YM, , 2015b. Placental lesions associated with acute atherosis. J. Matern Fetal Neonatal Med 28, 1554–1562. 10.3109/14767058.2014.960835 PMC4416076 25183023

[R67] Korzeniewski SJ, , 2016. Maternal plasma angiogenic index-1 (placental growth factor/soluble vascular endothelial growth factor receptor-1) is a biomarker for the burden of placental lesions consistent with uteroplacental underperfusion: a longitudinal case-cohort study. Am. J. Obstet. Gynecol 214, 629.e1–629.e17. 10.1016/j.ajog.2015.11.015 PMC5769706 26688491

[R68] Kupka E, , 2023. Low-dose aspirin use in pregnancy and the risk of preterm birth: a Swedish register-based cohort study. Am. J. Obstet. Gynecol 228, 336.e1–336.e9. 10.1016/j.ajog.2022.09.006 36096185

[R69] Kusanovic JP, , 2008. Twin-to-twin transfusion syndrome: an antiangiogenic state? Am. J. Obstet. Gynecol 198, 382.e1–382.e8. 10.1016/j.ajog.2008.02.016 PMC2678902 18395032

[R70] Kusanovic JP, , 2009. A prospective cohort study of the value of maternal plasma concentrations of angiogenic and anti-angiogenic factors in early pregnancy and midtrimester in the identification of patients destined to develop preeclampsia. J. Matern. Fetal Neonatal Med 22, 1021–1038. 10.3109/14767050902994754 PMC3427777 19900040

[R71] Labarrere CA, , 2017. Failure of physiologic transformation of spiral arteries, endothelial and trophoblast cell activation, and acute atherosis in the basal plate of the placenta. Am. J. Obstet. Gynecol 216, 287e1–287e16. 10.1016/j.ajog.2016.12.029 PMC5881902 28034657

[R72] Labarrere CA, , 2025. Evidence that atherosis of the spiral artery represents atherosclerotic lesions similar to those of native and transplant-induced atherosclerosis: implications for understanding the pathophysiology of obstetrical syndromes and long-term cardiovascular risk. 22:S0002–9378, (25) 00764–1. doi: 10.1016/j.ajog.2025.10.026. PMC13436850 41135844

[R73] Lekva T, , 2020. Multiplex analysis of circulating maternal cardiovascular biomarkers comparing preeclampsia subtypes. Hypertension 75, 1513–1522. 10.1161/HYPERTENSIONAHA.119.14580 32336238

[R74] Levine RJ, , 2004. Circulating angiogenic factors and the risk of preeclampsia. N. Engl. J. Med 350, 672–683. 10.1056/NEJMoa031884 14764923

[R75] Levine RJ, , 2006. Soluble endoglin and other circulating antiangiogenic factors in preeclampsia. N. Engl. J. Med 355, 992–1005. 10.1056/NEJMoa055352 16957146

[R76] Lin L, , 2022. A randomized controlled trial of low-dose aspirin for the prevention of preeclampsia in women at high risk in China. Am. J. Obstet. Gynecol 226, 251.e1–251.e12. 10.1016/j.ajog.2021.08.004 34389292

[R77] Malone SL, , 2022. Reviewing accuracy of first trimester screening for preeclampsia using maternal factors and biomarkers. Int. J. Women’s. Health 14, 1371–1384. 10.2147/IJWH.S283239 PMC9507456 36161188

[R78] Matthiesen L, , 2005. Immunology of preeclampsia. Chem. Immunol. Allergy 89, 49–61. 10.1159/000087912 16129952

[R79] Maynard SE, , 2003. Excess placental soluble fms-like tyrosine kinase 1 (sFlt1) may contribute to endothelial dysfunction, hypertension, and proteinuria in preeclampsia. J. Clin. Investig 111, 649–658. 10.1172/JCI17189 PMC151901 12618519

[R80] Mészáros B, , 2022. Pravastatin in preeclampsia: a meta-analysis and systematic review. Front. Med 9, 1076372. 10.3389/fmed.2022.1076372 PMC9880057 36714131

[R81] Mészáros B, , 2024. A meta-analysis on first-trimester blood count parameters-is the neutrophil-to-lymphocyte ratio a potentially novel method for first-trimester preeclampsia screening? Front. Med 11, 1336764. 10.3389/fmed.2024.1336764 PMC11021791 38633299

[R82] Moldenhauer JS, , 2003. The frequency and severity of placental findings in women with preeclampsia are gestational age dependent. Am. J. Obstet. Gynecol 189, 1173–1177. 10.1067/s0002-9378(03)00576-3 14586374

[R83] Moore MP, Redman CW, 1983. Case-control study of severe pre-eclampsia of early onset. Br. Med. J. (Clin. Res Ed 287, 580–583. 10.1136/bmj.287.6392.580 PMC1548969 6411232

[R84] Moser G, , 2018. Human trophoblast invasion: new and unexpected routes and functions. Histochem. Cell Biol 150, 361–370. 10.1007/s00418-018-1699-0 PMC6153604 30046889

[R85] Myatt L, Roberts JM, 2015. Preeclampsia: syndrome or disease? Curr. Hypertens. Rep 17, 83. 10.1007/s11906-015-0595-4 26362531

[R86] Nakashima A, , 2013. Impaired autophagy by soluble endoglin, under physiological hypoxia in early pregnant period, is involved in poor placentation in preeclampsia. Autophagy 9, 303–316. 10.4161/auto.22927 PMC3590252 23321791

[R87] Nelson DB, , 2014. Placental pathology suggesting that preeclampsia is more than one disease. Am. J. Obstet. Gynecol 210, 66.e1–66.e7. 10.1016/j.ajog.2013.09.010 24036400

[R88] Ness RB, Roberts JM, 1996. Heterogeneous causes constituting the single syndrome of preeclampsia: a hypothesis and its implications. Am. J. Obstet. Gynecol 175, 1365–1370. 10.1016/s0002-9378(96)70056-x 8942516

[R89] NICE, 2019. Hypertension in pregnancy: diagnosis and management. London. https://www.nice.org.uk/guidance/ng133. 31498578

[R90] Odibo AO, , 2015. Early prediction and aspirin for prevention of pre-eclampsia (EPAPP) study: a randomized controlled trial. Ultrasound Obstet. Gynecol 46, 414–418. 10.1002/uog.14889 25914193

[R91] Ogge G, , 2011. Placental lesions associated with maternal underperfusion are more frequent in early-onset than in late-onset preeclampsia. J. Perinat. Med 39, 641–652. 10.1515/JPM.2011.098 PMC3213694 21848483

[R92] Pallinger E, , 2023. Immune cell functionality during pregnancy. Orv. Hetil 164, 2006–2015. 10.1556/650.2023.32935 38142414

[R93] Papastefanou I, , 2020. Competing-risks model for prediction of small-forgestational-age neonate from maternal characteristics and medical history. Ultrasound Obstet. Gynecol 56, 196–205. 10.1002/uog.22129 32573831

[R94] Perry H, , 2018. Preeclampsia and the cardiovascular system: an update. Trends Cardiovasc. Med 28, 505–513. 10.1016/j.tcm.2018.04.009 29884568

[R95] Pijnenborg R, , 1983. Uteroplacental arterial changes related to interstitial trophoblast migration in early human pregnancy. Placenta 4, 397–413. 10.1016/s0143-4004(83)80043-5 6634666

[R96] Poon LC, , 2009. First-trimester prediction of hypertensive disorders in pregnancy. Hypertension 53, 812–818. 10.1161/HYPERTENSIONAHA.108.127977 19273739

[R97] Poon LC, , 2019. The international federation of gynecology and obstetrics (FIGO) initiative on pre-eclampsia: a pragmatic guide for first-trimester screening and prevention. Int. J. Gynaecol. Obstet 145 (1), 1–33. 10.1002/ijgo.12802 PMC6944283 31111484

[R98] Posta M, , 2024. The evolution of the Zinc Finger 554 (ZNF554) locus in anthropoid primates and its implications for deep placentation and preeclampsia. Placenta 154, e49.

[R99] Rana S, , 2012. Angiogenic factors and the risk of adverse outcomes in women with suspected preeclampsia. Circulation 125, 911–919. 10.1161/CIRCULATIONAHA.111.054361 PMC3319742 22261192

[R100] Rana S, , 2018. Angiogenic biomarkers in triage and risk for preeclampsia with severe features. Pregnancy Hypertens. 13, 100–106. 10.1016/j.preghy.2018.05.008 30177034

[R101] Rana S, , 2022. Imbalances in circulating angiogenic factors in the pathophysiology of preeclampsia and related disorders. Am. J. Obstet. Gynecol 226, S1019–S1034. 10.1016/j.ajog.2020.10.022 PMC8884164 33096092

[R102] Redline RW, , 2004. Maternal vascular underperfusion: nosology and reproducibility of placental reaction patterns. Pediatr. Dev. Pathol 7, 237–249. 10.1007/s10024-003-8083-2 15022063

[R103] Redline RW, , 2021. Four major patterns of placental injury: a stepwise guide for understanding and implementing the 2016 Amsterdam consensus. Mod. Pathol 34, 1074–1092. 10.1038/s41379-021-00747-4 33558658

[R104] Redman CW, , 2014. IFPA senior award lecture: making sense of pre-eclampsia - two placental causes of preeclampsia? Placenta 35, S20–S25. 10.1016/j.placenta.2013.12.008 24477207

[R105] Rezende KBC, , 2024. Performance of the first-trimester fetal medicine foundation competing risks model for preeclampsia prediction: an external validation study in Brazil. AJOG Glob. Rep 4, 100346. 10.1016/j.xagr.2024.100346 PMC11061323 38694483

[R106] Roberge S, , 2013. Prevention of perinatal death and adverse perinatal outcome using low-dose aspirin: a meta-analysis. Ultrasound Obstet. Gynecol 41, 491–499. 10.1002/uog.12421 23362106

[R107] Roberge S, , 2017. The role of aspirin dose on the prevention of preeclampsia and fetal growth restriction: systematic review and meta-analysis. Am. J. Obstet. Gynecol 216, 110–120.e6. 10.1016/j.ajog.2016.09.076 27640943

[R108] Roberge S, , 2018. Meta-analysis on the effect of aspirin use for prevention of preeclampsia on placental abruption and antepartum hemorrhage. Am. J. Obstet. Gynecol 218, 483–489. 10.1016/j.ajog.2017.12.238 29305829

[R109] Roberts JM, , 1989. Preeclampsia: an endothelial cell disorder. Am. J. Obstet. Gynecol 161, 1200–1204. 10.1016/0002-9378(89)90665-0 2589440

[R110] Roberts JM, , 2021. Subtypes of preeclampsia: recognition and determining clinical usefulness. Hypertension 77, 1430–1441. 10.1161/HYPERTENSIONAHA.120.14781 PMC8103569 33775113

[R111] Robillard PY, , 2019b. Increased BMI has a linear association with late-onset preeclampsia: a population-based study. PLOS One 14, e0223888. 10.1371/journal.pone.0223888 PMC6797165 31622409

[R112] Robillard PY, , 2019a. High incidence of early onset preeclampsia is probably the rule and not the exception worldwide. 20th anniversary of the reunion workshop. A summary. J. Reprod. Immunol 133, 30–36. 10.1016/j.jri.2019.05.003 31176084

[R113] Robillard PY, , 2022a. The blurring boundaries between placental and maternal preeclampsia: a critical appraisal of 1800 consecutive preeclamptic cases. J. Matern. Fetal Neonatal Med 35, 2450–2456. 10.1080/14767058.2020.1786516 32627713

[R114] Robillard PY, , 2022b. Progress in the understanding of the pathophysiology of immunologic maladaptation related to early-onset preeclampsia and metabolic syndrome related to late-onset preeclampsia. Am. J. Obstet. Gynecol 226, S867–S875. 10.1016/j.ajog.2021.11.019 35177223

[R115] Robillard PY, , 2024. Quantitative discrimination of small for gestational age (SGA) singleton newborns. incidences, risk factors and foetal outcomes of the three major subtypes of SGA: a 23-year cohort of 8,601 singleton SGA (Out of 83,917 Births). J. Pediatr. Perinatol. Child Health 8, 158–171.

[R116] Rolnik DL, , 2017. Aspirin versus placebo in pregnancies at high risk for preterm preeclampsia. N. Engl. J. Med 377, 613–622. 10.1056/NEJMoa1704559 28657417

[R117] Romero R, , 2008. A longitudinal study of angiogenic (placental growth factor) and anti-angiogenic (soluble endoglin and soluble vascular endothelial growth factor receptor-1) factors in normal pregnancy and patients destined to develop preeclampsia and deliver a small for gestational age neonate. J. Matern. Fetal Neonatal Med 21, 9–23. 10.1080/14767050701830480 PMC2587364 18175241

[R118] Romero R, 2009. Prenatal medicine: the child is the father of the man. J. Matern. Fetal Neonatal Med 22, 636–639. 10.1080/14767050902784171 19736614

[R119] Romero R, , 2010. An imbalance between angiogenic and anti-angiogenic factors precedes fetal death in a subset of patients: results of a longitudinal study. J. Matern. Fetal Neonatal Med 23, 1384–1399. 10.3109/14767051003681121 PMC3023956 20459337

[R120] Romero R, , 2022. Toward a new taxonomy of obstetrical disease: improved performance of maternal blood biomarkers for the great obstetrical syndromes when classified according to placental pathology. Am. J. Obstet. Gynecol 227, 615.e1–615.e25. 10.1016/j.ajog.2022.04.015 PMC9525890 36180175

[R121] Saito S, Nakashima A, 2014. A review of the mechanism for poor placentation in early-onset preeclampsia: the role of autophagy in trophoblast invasion and vascular remodeling. J. Reprod. Immunol 101-102, 80–88. 10.1016/j.jri.2013.06.002 23969229

[R122] Salahuddin S, , 2016. KRYPTOR-automated angiogenic factor assays and risk of preeclampsia-related adverse outcomes. Hypertens. Pregnancy 35, 330–345. 10.3109/10641955.2016.1148162 27028795

[R123] Savasan ZA, , 2010. Evidence in support of a role for anti-angiogenic factors in preterm prelabor rupture of membranes. J. Matern. Fetal Neonatal Med 23, 828–841. 10.3109/14767050903440471 PMC3104674 20158393

[R124] Scioscia M, , 2015. Endothelial dysfunction and metabolic syndrome in preeclampsia: an alternative viewpoint. J. Reprod. Immunol 108, 42–47. 10.1016/j.jri.2015.01.009 25766966

[R125] Sibai B, , 2005. Pre-eclampsia. Lancet 365, 785–799. 10.1016/S0140-6736(05)17987-2 15733721

[R126] Sinha N, , 2023. A randomized controlled study comparing the efficacy of 75mg Versus 150mg aspirin for the prevention of preeclampsia in high-risk pregnant women. Cureus 15, e39752. 10.7759/cureus.39752 PMC10311037 37398778

[R127] Soto E, , 2012. Late-onset preeclampsia is associated with an imbalance of angiogenic and anti-angiogenic factors in patients with and without placental lesions consistent with maternal underperfusion. J. Matern. Fetal Neonatal Med 25, 498–507. 10.3109/14767058.2011.591461 PMC3401571 21867402

[R128] Sovio U, , 2017. Prediction of preeclampsia using the soluble fms-like tyrosine kinase 1 to placental growth factor ratio: a prospective cohort study of unselected nulliparous women. Hypertension 69, 731–738. 10.1161/HYPERTENSIONAHA.116.08620 PMC5344177 28167687

[R129] Staff AC, , 2010. Learning from the placenta: acute atherosis and vascular remodeling in preeclampsia-novel aspects for atherosclerosis and future cardiovascular health. Hypertension 56, 1026–1034. 10.1161/HYPERTENSIONAHA.110.157743 20956732

[R130] Staff AC, , 2013. Review: Preeclampsia, acute atherosis of the spiral arteries and future cardiovascular disease: two new hypotheses. Placenta 34, S73–S78. 10.1016/j.placenta.2012.11.022 23246096

[R131] Staff AC, , 2022. Failure of physiological transformation and spiral artery atherosis: their roles in preeclampsia. Am. J. Obstet. Gynecol 226, S895–S906. 10.1016/j.ajog.2020.09.026 32971013

[R132] Staff AC, Redman CW, 2014. IFPA award in placentology lecture: preeclampsia, the decidual battleground and future maternal cardiovascular disease. Placenta 35, S26–S31 (Suppl). 10.1016/j.placenta.2013.12.003 24411701

[R133] Stanek J, 2014. Comparison of placental pathology in preterm, late-preterm, near-term, and term births. Am. J. Obstet. Gynecol 210, 234.e1–234.e6, 234 e1–6. 10.1016/j.ajog.2013.10.015 24145185

[R134] Sundrani D, , 2013. Gestation dependant changes in angiogenic factors and their associations with fetal growth measures in normotensive pregnancy. PLOS One 8, e54153. 10.1371/journal.pone.0054153 PMC3542273 23326589

[R135] Surányi A, , 2023. Correlation between placental perfusion measured in the entire placenta versus assessed by sonobiopsy. Orv. Hetil 164, 300–307. 10.1556/650.2023.32702 36842147

[R136] Szabo S, , 2015. Activation of villous trophoblastic p38 and ERK1/2 signaling pathways in preterm preeclampsia and HELLP syndrome. Pathol. Oncol. Res 21, 659–668. 10.1007/s12253-014-9872-9 PMC5987200 25583406

[R137] Szalai G, , 2014. In vivo experiments reveal the good, the bad and the ugly faces of sFlt-1 in pregnancy. PLOS One 9, e110867. 10.1371/journal.pone.0110867 PMC4230935 25393290

[R138] Szalai G, , 2015. Full-length human placental sFlt-1-e15a isoform induces distinct maternal phenotypes of preeclampsia in mice. PLOS One 10, e0119547. 10.1371/journal.pone.0119547 PMC4393117 25860260

[R139] Thadhani R, , 2004. First trimester placental growth factor and soluble fms-like tyrosine kinase 1 and risk for preeclampsia. J. Clin. Endocrinol. Metab 89, 770–775. 10.1210/jc.2003-031244 14764795

[R140] Than GN, , 1993. Advances in pregnancy-related protein research. Functional and clinical applications. CRC Press: Boca Raton, FL, USA. https://www.taylorfrancis.com/books/edit/10.1201/9780367812164/advances-pregnancy-related-protein-research-functional-clinical-applications-denes-szabo-gabor-bohn.

[R141] Than NG, , 2018. Integrated systems biology approach identifies novel maternal and placental pathways of preeclampsia. Front. Immunol 9, 1661. 10.3389/fimmu.2018.01661 PMC6092567 30135684

[R142] Than NG, , 2022. Early pathways, biomarkers, and four distinct molecular subclasses of preeclampsia: the intersection of clinical, pathological, and high-dimensional biology studies. Placenta 125, 10–19. 10.1016/j.placenta.2022.03.009 PMC9261837 35428514

[R143] Than NG, , 2023. Molecular subclasses of preeclampsia characterized by a longitudinal maternal proteomics study: distinct biomarkers, disease pathways and options for prevention. J. Perinat. Med 51, 51–68. 10.1515/jpm-2022-0433 PMC9837387 36253935

[R144] Than NG, , 2024. Classification of preeclampsia according to molecular clusters with the goal of achieving personalized prevention. J. Reprod. Immunol 161, 104172. 10.1016/j.jri.2023.104172 PMC11027116 38141514

[R145] Tsikouras P, , 2024. Diagnosis, prevention, and management of fetal growth restriction (FGR). J. Pers. Med 14. 10.3390/jpm14070698 PMC11278205 39063953

[R146] Vaisbuch E, , 2011. Circulating angiogenic and antiangiogenic factors in women with eclampsia. Am. J. Obstet. Gynecol 204, 152.e1–152.e9. 10.1016/j.ajog.2010.08.049 PMC3057127 21062661

[R147] Varkonyi T, , 2011. Microarray profiling reveals that placental transcriptomes of early-onset HELLP syndrome and preeclampsia are similar. Placenta 32, S21–S29 (Suppl). 10.1016/j.placenta.2010.04.014 PMC3917714 20541258

[R148] Venkatesha S, , 2006. Soluble endoglin contributes to the pathogenesis of preeclampsia. Nat. Med 12, 642–649. 10.1038/nm1429 16751767

[R149] Verlohren S, , 2010. An automated method for the determination of the sFlt-1/PIGF ratio in the assessment of preeclampsia. Am. J. Obstet. Gynecol 202, 161.e1–161.e11. 10.1016/j.ajog.2009.09.016 19850276

[R150] Verlohren S, , 2014. Uterine artery Doppler, birth weight and timing of onset of pre-eclampsia: providing insights into the dual etiology of late-onset pre-eclampsia. Ultrasound Obstet. Gynecol. 44, 293–298. 10.1002/uog.13310 24448891

[R151] Verma ML, , 2022. Placental growth factor in first trimester of pregnancy for prediction of maternal and perinatal adverse outcomes. J. Obstet. Gynaecol. India 72, 396–401. 10.1007/s13224-022-01636-9 PMC9568639 36458060

[R152] Villa PM, , 2013. Aspirin in the prevention of pre-eclampsia in high-risk women: a randomised placebo-controlled PREDO trial and a meta-analysis of randomised trials. BJOG 120, 64–74. 10.1111/j.1471-0528.2012.03493.x 23126307

[R153] Whitten AE, , 2013. Evidence of an imbalance of angiogenic/antiangiogenic factors in massive perivillous fibrin deposition (maternal floor infarction): a placental lesion associated with recurrent miscarriage and fetal death. Am. J. Obstet. Gynecol 208, 310.e1–310.e11. 10.1016/j.ajog.2013.01.017 PMC3939787 23333548

[R154] Windsperger K, , 2017. Extravillous trophoblast invasion of venous as well as lymphatic vessels is altered in idiopathic, recurrent, spontaneous abortions. Hum. Reprod 32, 1208–1217. 10.1093/humrep/dex058 28369440

[R155] Zeisler H, , 2019. Soluble fms-like tyrosine kinase-1 to placental growth factor ratio: ruling out pre-eclampsia for up to 4 weeks and value of retesting. Ultrasound Obstet. Gynecol 53, 367–375. 10.1002/uog.19178 PMC6590225 30014562

[R156] Zhang L, , 2023. Unraveling the mysteries of spiral artery remodeling. Placenta 141, 51–56. 10.1016/j.placenta.2023.05.013 37308346

[R157] Zwertbroek EF, , 2021. Performance of the FMF first-trimester preeclampsia-screening algorithm in a high-risk population in The Netherlands. Fetal Diagn. Ther 48, 103–111. 10.1159/000512335 33401268

